# Quantitative Detection of the Foot-And-Mouth Disease Virus Serotype O 146S Antigen for Vaccine Production Using a Double-Antibody Sandwich ELISA and Nonlinear Standard Curves

**DOI:** 10.1371/journal.pone.0149569

**Published:** 2016-03-01

**Authors:** Xia Feng, Jun-Wu Ma, Shi-Qi Sun, Hui-Chen Guo, Ya-Min Yang, Ye Jin, Guang-Qing Zhou, Ji-Jun He, Jian-Hong Guo, Shu-yun Qi, Mi Lin, Hu Cai, Xiang-Tao Liu

**Affiliations:** State Key Laboratory of Veterinary Etiological Biology, OIE/National Foot-and-Mouth Disease Reference Laboratory, Lanzhou Veterinary Research Institute, Chinese Academy of Agricultural Sciences, Lanzhou, Gansu, The People’s Republic of China; Institut National de la Santé et de la Recherche Médicale (INSERM), FRANCE

## Abstract

The efficacy of an inactivated foot-and-mouth disease (FMD) vaccine is mainly dependent on the integrity of the foot-and-mouth disease virus (FMDV) particles. At present, the standard method to quantify the active component, the 146S antigen, of FMD vaccines is sucrose density gradient (SDG) analysis. However, this method is highly operator dependent and difficult to automate. In contrast, the enzyme-linked immunosorbent assay (ELISA) is a time-saving technique that provides greater simplicity and sensitivity. To establish a valid method to detect and quantify the 146S antigen of a serotype O FMD vaccine, a double-antibody sandwich (DAS) ELISA was compared with an SDG analysis. The DAS ELISA was highly correlated with the SDG method (R^2^ = 0.9215, P<0.01). In contrast to the SDG method, the DAS ELISA was rapid, robust, repeatable and highly sensitive, with a minimum quantification limit of 0.06 μg/mL. This method can be used to determine the effective antigen yields in inactivated vaccines and thus represents an alternative for assessing the potency of FMD vaccines *in vitro*. But it still needs to be prospectively validated by analyzing a new vaccine preparation and determining the proper protective dose followed by an *in vivo* vaccination-challenge study to confirm the ELISA findings.

## Introduction

Foot-and-mouth disease (FMD) is one of the most economically significant trans-boundary diseases among animals; this condition causes severe production losses in domesticated and wild cloven-hoofed animals, particularly in the dairy and pig industries [1]. FMD viruses (FMDV) can be divided into seven immunologically distinct serotypes: O, A, C, Asia 1, SAT 1, SAT 2 and SAT 3. Infection with any one serotype does not produce immunity against another serotype. The three most prevalent serotypes in Asia are O, A and Asia 1 [2, 3], while the SAT-1 thru SAT-3 serotypes are mainly restricted to sub-Saharan Africa [4].

Vaccination is one of the most practical and effective measures to prevent outbreaks of FMD [5]. Inactivated whole-virus vaccines are produced from cell cultures infected with FMDV and are the most widely used vaccines in China. However, the use of these vaccines requires strict control of the antigen quality (such as tests for 146S quantification, sterility, identity, purity, safety, potency, stability and immunity) [6–8].

The current method for testing the potency of FMD vaccines is the challenge test, which is performed in the target species. To date, the “Gold Standard” test has been the *in vivo* challenge of primo-vaccinated animals. Two direct methods are commonly used in testing: the 50% protective dose (PD_50_) test and the South-American Protection against Generalization (PG) test [6, 7]. The traditional method has proven to play a very important role in developing and controlling FMD vaccines. However, the challenge experiments have several drawbacks, including high variability, high cost, a significant time requirement, a requirement for facilities with high biosecurity levels and the use of a large number of animals; thus, the standardization of the experiments is not easy. Official animal health services and experts from *the Office International des Epizootie*s (OIE) have supported the use of alternative reliable indirect methods to replace the vaccine potency experiments. These alternative methods are mainly divided into the following three categories: serological methods [8–14], laboratory animal methods [15–17] and *in vitro* quantitative methods to assess antigens [18–24]. Compared with the former two methods, the *in vitro* quantification of FMD whole virus particles is more convenient and can be performed at any time during vaccine production.

Based on sedimentation coefficients, FMDV can be divided into four specific particles using sucrose gradient centrifugation: intact virions (146S or 140S), empty capsids (75S), virus infection-related peptides (45S) and 12S protein subunits (12S). The efficacy of inactivated vaccines is mainly dependent on the integrity of the FMDV particles (146S) [25– 28].

The 146S quantitative sucrose density gradient centrifugation (SDG) technique developed by Barteling and Meloen (1975) is the recommended method to quantify virus antigens [18]. Over the past 40 years, SDG has proven to be a reliable method for the measurement of virus concentration. However, this method is not only labor-intensive and time-consuming, but SDG also requires expensive specialized equipment and is highly operator dependent. A number of international efforts to standardize this method have been attempted; however, there is neither a uniform protocol nor an international standard [19]. Moreover, the variability of SDG procedures performed in different laboratories is too great to use this method to assess FMD vaccine products from different manufacturers.

Alternatively, an enzyme-linked immunosorbent assay (ELISA) offers greatly decreased assay times as well as increased simplicity and sensitivity. However, immunoassay methods for the quantification of 146S are not easy because the whole virus and its subunit (12S) share many of the same epitopes, and most monoclonal and polyclonal antibodies directed against FMDV are cross-reactive to both 146S and 12S [26–27,29]. However, the specificity of monoclonal antibodies (MAbs) for whole virus particles enables the development of serological systems that can overcome the problems of cross-reactivity [20–23].

In the present study, we combined, for the first time, SDG with an ELISA and used nonlinear standard curves to develop a double-antibody sandwich (DAS) ELISA using polyclonal antibodies to quantify the serotype O FMDV 146S antigen. There was a strong correlation between the concentrations of 146S antigen obtained with this test and those found using the SDG method. In contrast to the SDG method, the DAS ELISA can simultaneously quantify intact antigens in a large number of samples.

## Materials and Methods

### Standard Antigen and Inactivated Vaccines

FMDV vaccine strains O/MYA98/BY/2010 (GenBank JN998085) and O/China/99 (GenBank AF506822) were obtained from China Agricultural Vet. Boi. Science and Technology Co., Ltd. Viruses were propagated at industrial scale in baby hamster kidney cells 21 (BHK-21) [30] and inactivated with 0.01 M binary ethylenimine (BEI) [31]. Viral antigens were centrifuged for 0.5 h at 4°C, 11 260 g, and were stored at -70°C either in 5-mL aliquots or 4×100-mL aliquots (only freezing and thawing one time). The former series of aliquots were used as standard antigen in ELISA tests and the latter for virus purification in SDG. Inactivated antigen and vaccine samples were provided by vaccine manufacturers.

### Preparation of Samples

**Fig 1:**
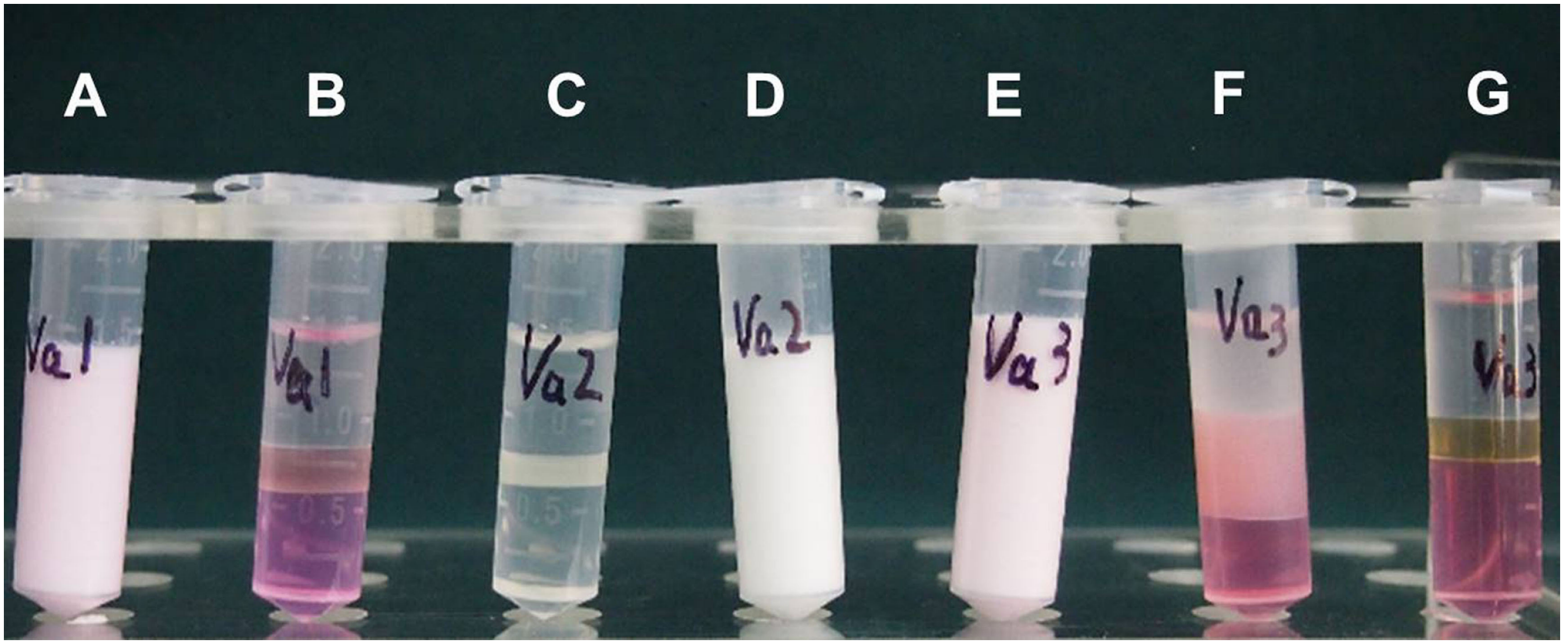
Extract antigen from oil emulsion vaccine. Antigens from oil emulsion vaccines were recovered from the aqueous phases after the rupture of the water-in-oil emulsions with n-butyl alcohol. Briefly, nine volumes of vaccine were thoroughly mixed with one volume of n-butyl alcohol and centrifuged for 5 min at 4°C, 5,000 g. The lower aqueous phases were collected carefully and used for subsequent antigen quantification. (A) Vaccine No. 1. (B) Vaccine No. 1 mixed with n-butyl alcohol and breaking of the emulsion complete. (D) Vaccine No. 2. (C) Vaccine No. 2 mixed with n-butyl alcohol and breaking of the emulsion complete. (E) Vaccine No. 3. (F) Vaccine No. 3 mixed with n-butyl alcohol and breaking of the emulsion incomplete, the intermediate layer was not clear and the volume of the aqueous layer was quite small. (G) Vaccine No. 3 mixed with n-butyl alcohol complete (by increasing the amount of n-butyl alcohol).

Inactivated antigens will be used directly. The live virus was obtained by freezing and thawing for three times to let the virus release completely. Antigens from oil emulsion vaccines were recovered from the aqueous phases after the rupture of the water-in-oil emulsions with n-butyl alcohol. Briefly, nine volumes of vaccine were thoroughly mixed with one volume of n-butyl alcohol and centrifuged for 5 min at 4°C, 5,000 g. The lower aqueous phases were collected carefully and used for subsequent antigen quantification (Fig 1).

### Sucrose Gradient Centrifugation

The traditional SDG method was performed as previously described with some modified [18]. Briefly, standard antigen was degreased with pre-cold trichloroethylene (4°C). The upper aqueous was precipitated by ultracentrifugation at 136 000 g at 4°C for 150 min by an Optima L-100 XP ultracentrifuge (Beckman Coulter, CA, USA). The pellet was suspended by the grinding suspension device (China Patent No. 201420465197.1) with TNE Buffer (10mM Tris, 150Mm NaCl, 1mM EDTA, pH 7.6) [32]. The 146S antigen was purified from others by separating them on linear 15–45% sucrose density gradients after centrifugation at 110 000 g at 8°C for 180 min. Each fraction from such gradient was measured at a wavelength of 259 nm. The 146S antigen amount was determined. Standard antigen was purified and quantified three times and the other test samples were purified and quantified only once.

### DAS ELISA

Anti-FMDV O 146S antigen antiserum was obtained by mixing fresh FMDV O 146S antigen fractions with Freund’s complete adjuvant and injecting the mixture into rabbits and guinea pigs. After 28 days, all of the rabbits and guinea pigs were bled out, and the antisera were collected. All animal experiments were carried out according to the protocols approved by the Institutional Committee for Animal Care and the policy of the National Ministry of Health. For the ELISA assay, 96-well ELISA plates (Costar, Corning, NY, USA) were coated with the rabbit anti-FMDV O 146S antigen antisera diluted 1:6,000 in 0.05 M carbonate/ bicarbonate buffer (pH 9.6) (Sigma-Aldrich, St. Louis, MO, USA) and incubated overnight at 4°C. FMDV standard antigen and antigen samples were diluted in a twofold dilution series across the plates (in a parallel line) in antigen diluent buffer (phosphate-buffered saline (PBS) containing 0.05% Tween 20 and 2% bovine serum albumin). Guinea pig antiserum was prepared (1:3,000) in blocking buffer (PBS containing 0.05% Tween 20, 10% normal bovine serum and 5% normal rabbit serum). HRP-conjugated goat anti-guinea pig IgG (Sigma-Aldrich, St. Louis, MO, USA) was added to each well. ortho-Phenylenediamine /hydrogen peroxide (OPD, Sigma-Aldrich, St. Louis, MO, USA) was used as the substrate. Sulfuric acid (1.0 M) was added to stop the color reaction. The absorbance was measured at 490 nm with an automated ELISA reader (BIO-RAD 680, Japan). All of the incubation steps were performed for 60 min at 37°C, followed by four washes with washing buffer (PBS containing 0.05% Tween 20; PBST), except for the final incubation, which was 15 min at 37°C with no washing step. The OD values were sent to the pre-programmed EXCEL file to calculate the antigen contents of the samples using the standard curves (based on Curve Expert 1.4, Daniel G. Hyams).

The nonlinear standard curve was derived from the FMDV antigen reference standard, which was serially diluted in the blocking buffer at a ratio of 1:2 to obtain the following 146S antigen concentrations: 1.0, 0.5, 0.25, 0.125, 0.0625, 0.03125, 0.0156 and 0.0078 μg/mL, in accordance with the professional software “Curve Expert 1.4”. This process was used to estimate the concentration of the unknown samples. The assay was only accepted if the coefficients of variation (CVs) of the duplicates were less than 10% and the correlation coefficient of the standard curve was greater than 0.999.

Nonlinear standard curve was constructed according to polynomial regression Eq (1) (degree = 4–6): where x is the absorbance at 490 nm, y is the concentration of the antigen reference standard in μg/mL, and a, b, c, d, e, f… are the parameters.

$$y=a+bx+cx^{2}+dx^{3}+ex^{4}+fx^{5}+\cdots$$ (1)

### Evaluation of the LD (Limit of Detection), LQ (Limit of Quantitation), Precision, Linearity of Dilution and Specificity

The blank controls (BHK-21 cells) were assayed 20 times in duplicate. The LD was evaluated by interpolating the mean of 40 values plus 10 standard deviations.

The LQ was calculated by adding the LD to the highest value obtained from the Asia 1/JS (GenBank EF149009) sample, which was higher than the value obtained from the A/HuBWH (GenBank JF792355).

To assess the intra-assay precision and inter-assay precision, ten FMDV samples, including four vaccine samples, three antigen samples and three virus samples, were assayed in 5 replicates within the plate. Each sample was assayed nine times to determine the precision within an assay and between assays.

The linearity of dilution was evaluated by serially diluting 4 test samples. The linearity of dilution was calculated based on the concentration results of the 3–5 dilutions determined from the standard curve using the pre-programmed EXCEL procedures. The calculated concentration results were compared with the expected concentrations.

The specificity of the DAS ELISA was analyzed using a dilution series of the following FMDV strain antigens: O/MYA98/BY/2010, O/China/99, Asia 1/JS, and A/HuBWH. The antigens were tested from a constant high initial amount (2.0 μg/mL). Blank samples (BHK-21 cells and PBS) and other disease antigens, such as swine vesicular disease virus (SVDV, GenBank AY429470), classical swine fever virus (CSFV, GenBank AY805221), porcine reproductive and respiratory syndrome virus (PRRSV, GenBank GU047345) and bovine viral diarrhea virus (BVDV, GenBank KC297709) were also included.

### Correlation of the DAS ELISA with SDG

To assess the reliability of the 146S antigen content obtained using this method, a comparison was performed between the DAS ELISA and SDG methods. For this purpose, eighty-five samples including live FMDV (n = 17, 39–55), samples of the inactivated virus preparation (n = 50, 1–20, 56–85) and vaccine samples (n = 18, 21–38) were tested with both methods. The results were compared using the software “Curve Expert 1.4”.

### The DAS ELISA Comparison Test

Six batches of type O vaccine from different companies (listed in Table 1) were randomly coded and tested. The technical staff of the above companies was divided into three groups and requested to follow the DAS ELISA protocol to test the samples. The results were calculated and compared with the SDG values. The %CV for the intra-group assays was also calculated.

**Table 1:** FMD vaccine manufacturers.

	Names	Vaccine antigen
1	CAHIC ^a^ Lanzhou Biological Pharmaceutical companies	★ ^b^
2	China Agricultural Vet. Boi. Science and Technology Co., Ltd.	★ ^b^; ▼^c^
3	CAHIC ^a^ Baoshan Biological Pharmaceutical companies	★ ^b^
4	the Spirit Jinyu Biological Pharmaceutical Co., Ltd	▼ ^c^
5	Xinjiang Tiankang Animal Science Bio-Technology Co., Ltd	★ ^b^
6	BIGVET = China BIGVET Group Bio-Technology Co., Ltd.	★ ^b^

^a^ CAHIC = China Animal Husbandry Industry Co., Ltd;

^b^**★** = crude antigen;

^c^▼ = half concentrated antigen

### Ethics Statement

All animals received humane care in compliance with good animal practice according to the Animal Ethics Procedures and Guidelines of the People's Republic of China. The animal experiments were carried out according to the protocols approved by the Institutional Committee of Lanzhou Veterinary Research Institute, Chinese Academy of Agricultural Sciences (permit number LVRIAEC2013-014, -015).

### Statistical Analysis

The variations within an assay and between assays were calculated by One-way Analysis of Variance (ANOVA) [33].

## Results

### Quantification of the FMDV Standard Antigens

The 146S particle standard antigens were purified and quantified three times by SDG to obtain a final concentration of 2.0 μg/mL (O/MYA98: 1.9826±0.255, %CV = 12.87; O/China/99: 2.022±0.305, %CV = 15.08). We abandoned the precipitation step with polyethylene glycol (PEG) 6000 during the purification process because the yields of the 146S antigen were reduced by at least 15% using this method, and a previous study also found that PEG precipitation resulted in only a 79.2% recovery of poliovirus infectivity [34]. Furthermore, we selected the grinding suspension device to suspend the precipitates after ultracentrifugation using a cold chain system to inhibit 146S antigen degradation [32]. Using this method, the precipitates are not easily lost during the process of suspension. The grinding suspension device is thus very useful for quantification.

### Evaluation of the DAS ELISA

#### LD and LQ

The LD was evaluated by interpolating the mean of 40 values obtained with BHK-21 cells (20 tests in duplicate) plus 10 standard deviations. The LD was 0.155 (0.065+0.009×10 = 0.155) (Table A in S1 File).

The LQ was calculated using the LD (0.155) plus the highest value obtained from the Asia 1/JS sample (0.311), which was higher than the value obtained from the A/HuBWH serotype (0.21). The LQ was 0.466 (0.155+0.311 = 0.466).

The lower limit of quantitation (LLQ) was 0.06 μg/mL (Table B in S1 File). There was no upper limit of quantitation (ULQ); if the concentration of the sample could not be calculated because the OD_490_ of the test sample was too high even at the last dilution, continuous serial dilutions were performed over an even wider range until the original concentration of the sample could be calculated by the measurement of concentrations corresponding to several serial dilutions.

#### Precision

**Fig 2:**
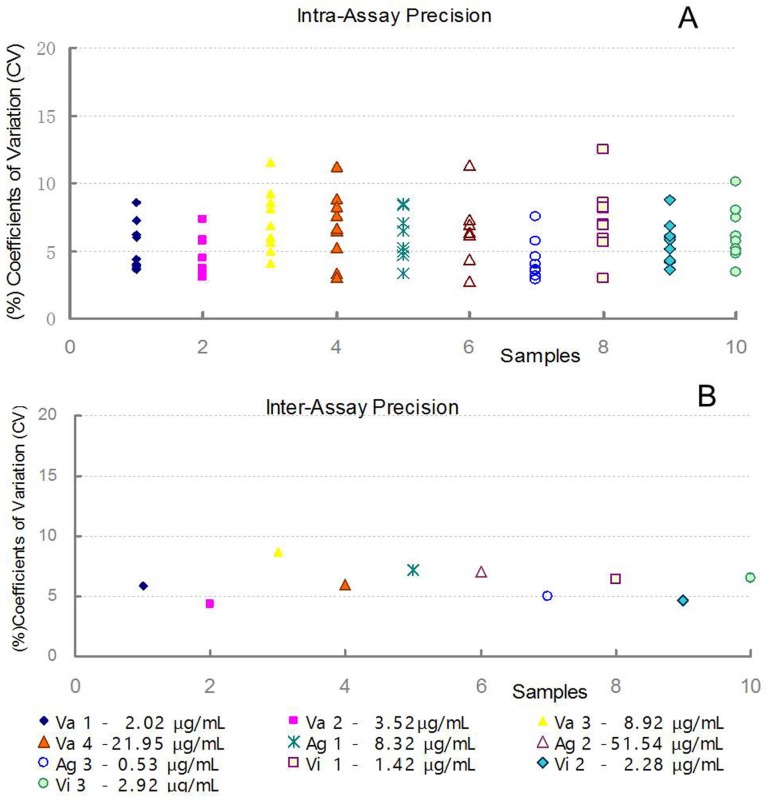
The intra-assay precision (A, 2.87–12.43%) and the inter-assay precision (B, 4.33–8.67%) of the DAS ELISA. Ten FMDV samples, including 4 vaccine samples (V1-V4), 3 antigen samples (Ag1-Ag3) and 3 virus samples (B1-B3), were assayed 9 times to determine precision within an assay and between assays, the coefficients of variations (CV, %) were plotted and the means of the samples were showed.

In this study, ten FMDV samples were assayed nine times to assess the precision within an assay and between assays. The data were tested by Bonferroni’s Multiple Comparison Test (one-way ANOVA), ploting a frequency distribution by using GraphPad Prism 5 software (GraphPad software inc., CA, USA). For all analyses, P < 0.05 was considered significant. The intra-assay variation was 2.87–12.43% (0.0504≤p≤0.237) and the variation between assays was 4.33–8.67% (p = 0.0772, Fig 2, Table C in S1 File).

#### Linearity of dilution

The linearity of dilution results for four samples was shown in Table 2. The calculated concentration results versus the expected concentrations yielded an average correlation coefficient of 0.98.

**Table 2:** Linearity of dilution: the measured concentration of 4 samples versus the expected concentration at certain dilutions.

sample 1 (ng/mL)				sample 2 (ng/mL)			
Dilution	Measured	Expected	% Expected	Dilution	Measured	Expected	% Expected
1/1	8 343.81	/	/	1/1	21 392.90	/	/
1/16	462.00	521.49	/	1/16	1 364.36	1 337.06	102.0
1/32	256.65	260.74	98.4	1/32	623.38	668.53	93.2
1/64	132.49	130.37	101.6	1/64	348.90	334.26	104.4
1/128	63.91	65.19	98.0	1/128	167.69	167.13	100.3
1/256	33.21	32.59	101.9	1/256	90.08	83.57	107.8
Sample3 (ng/mL)				sample 4 (ng/mL)			
Dilution	Measured	Expected	% Expected	Dilution	Measured	Expected	% Expected
1/1	986.37	/	/	1/1	2 501.31	/	/
1/8	119.96	123.30	97.3	1/8	2 97.94	312.66	95.3
1/16	55.86	61.65	90.6	1/16	160.79	156.33	102.8
1/32	34.89	30.83	113.2	1/32	78.11	78.17	99.9
1/64	18.39	15.41	119.3	1/64	39.83	39.08	101.9

#### Specificity

**Fig 3:**
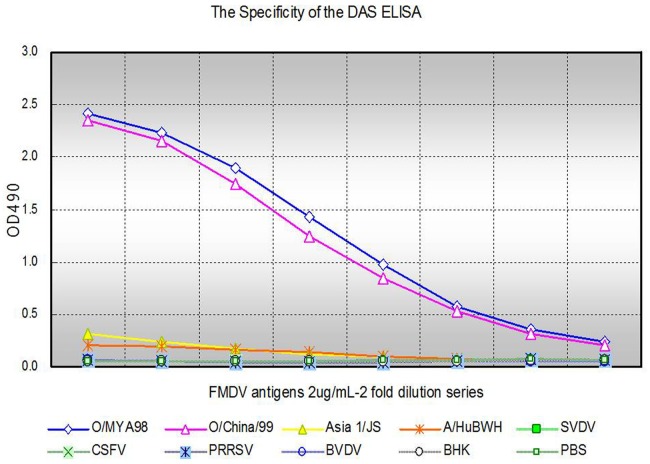
The specificity of the DAS ELISA. The specificity of the DAS ELISA was analysed through dilution series of the following FMDV strain antigens: O/MYA98, O/China/99, Asia 1/JSL and A/HuBWH serotypes, from a constant high initial amount (2.0 μg/mL), and the blank samples of BHK-21 cells and PBS, as well as the other disease antigens, such as: swine vesicular disease virus (SVDV), classical swine fever virus (CSFV), porcine reproductive and respiratory syndrome virus (PRRSV) and bovine viral diarrhoea virus (BVDV). The results indicated that the O/MYA98 and O/China/99 can be specifically detected among the others by the absorbance values obtained for testing.

The DAS ELISA proved to have a high specificity. No reactions were detected with BHK-21 cells, PBS, SVDV, CSFV, BVDV or PRRSV, and negative reactions were also obtained with the other FMDV strains. However, the assay produced a strong positive reaction with the serotype O strains (Fig 3, Table D in S1 File).

### 146S Antigen Quantification via Both DAS ELISA and SDG

**Fig 4:**
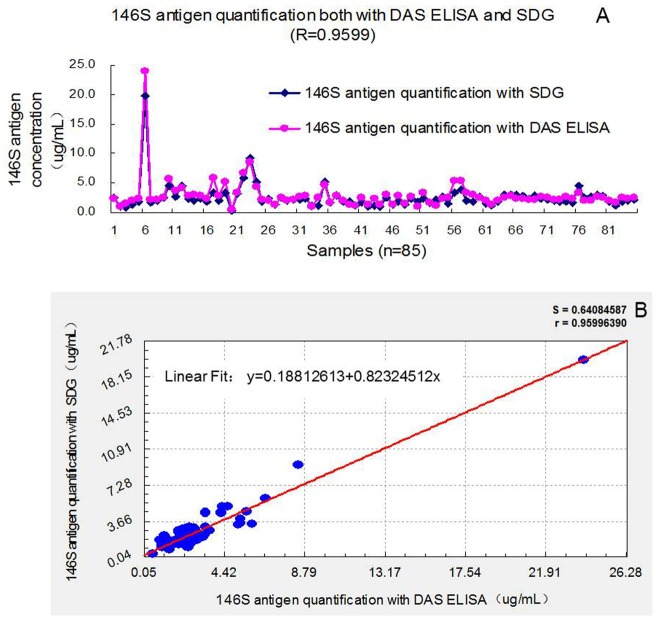
Comparison of the 146S antigen quantification by the DAS ELISA and SDG method. 85 FMDV samples, including FMDV live (n = 17, 39^#^-55^#^), inactivated antigen samples (n = 50,1^#^-20^#,^57^#^-85^#^) and vaccine samples (n = 18, 21^#^-38^#^), were tested with both methods. The results were compared using the software “Curve Expert 1.4”, and it demonstrated that the DAS ELISA was highly correlated with the traditional SDG method (R^2^ = 0.9215, P<0.01).

We analyzed 85 samples using both the DAS ELISA and SDG methods, and the results (Table E in S1 File) demonstrated that the DAS ELISA was highly correlated with the SDG method (R^2^ = 0.9215, P<0.01) (Fig 4). Thus, the performance of the DAS ELISA assay was demonstrated to be concordant with the traditional SDG method for the quantification of virus particles. However, the DAS ELISA exhibited a detection limit of 0.06 μg/mL antigen and thus was more sensitive than the SDG method, which had a detection limit of 0.6–0.8 μg/mL. These data demonstrated that the DAS ELISA is a reliable and sensitive method for the quantification of the 146S antigen, even at low quantities of antigen.

### The DAS ELISA Comparison Test

The six batches of type O vaccine samples were tested by three groups using the DAS ELISA protocol. The results (Table 3) obtained were relatively stable (%CV 2.80–13.75) and had a strong correlation with the traditional SDG values that were provided by the vaccine manufacturers (R^2^ = 0.8310), with the exception of two samples (O-5 and O-6) that could not easily be get the aqueous phases from the water-in-oil emulsions. In these cases, the %CV for the intra-group assays for these two samples had a comparatively wide variation (35.86–18.92). Therefore, the antigen recovery from oil-emulsion vaccines is critical.

**Table 3:** Vaccine samples tested in the ELISA comparison test.

	146S antigen concentration measured by (μg/mL)						
samples	SDG	the DAS ELISA					
	providing by manufactures	Group 1	Group 2	Group 3	Mean	SD	%CV
O-1	1.10	2.586	2.417	2.480	2.494	0.070	2.802
O-2	5.13	5.001	3.735	5.157	4.631	0.637	13.751
O-3	no data	1.633	1.755	1.943	1.777	0.127	7.173
O-4	1.71	1.931	1.489	1.651	1.691	0.182	10.792
O-5 ^a^	2.86	2.095	2.177	4.311	2.861	1.026	35.858
O-6 ^a^	1.89	1.593	1.850	2.482	1.975	0.374	18.919

^a^ Antigens from O-5 and O-6 were not easily be recovered from the two emulsions because of incompletely rupture with n-butyl alcohol.

## Discussion

In the present study, we developed a DAS ELISA based on an ELISA and SDG using nonlinear standard curves and polyclonal antibodies for the quantitative detection of the FMDV 146S antigen in live/inactive viral antigen and oil-emulsion vaccines.

It is very important to select the most appropriate standard for optimal DAS ELISA performance. The FMD vaccines consisted of either crude inactive viral antigens or half concentrated antigens; therefore, crude inactive viral antigens were chosen as the standard antigen in this study. These antigens were composed of a mixture of substances that were as similar as possible to the unknown FMDV samples. To ensure that the standards were consistent throughout the entire investigation, a large batch of the standards was prepared and stored in aliquots at -70°C (using each only once). Additionally, nonlinear standard curves were used instead of classical linear curves. These experimental conditions ensured that our method possessed more advantages than the traditional antigen quantification method.

The 146S antigen was purified from the 12S virus protein subunits, the VIA antigen and most of the cell debris with the SDG method, and only the value of this component of the standard antigen was used to determine the 146S antigen content of the tested samples. Double repeat serial dilutions of the antigen reference standard were included on each ELISA plate to generate a standard curve and to avoid bias when comparing the test samples between different plates. In this study, the standard antigen served not only as a “ruler” but also as a “bridge” between the SDG and ELISA methods. Therefore, the selection of the standard antigen is crucial for our method.

**Fig 5:**
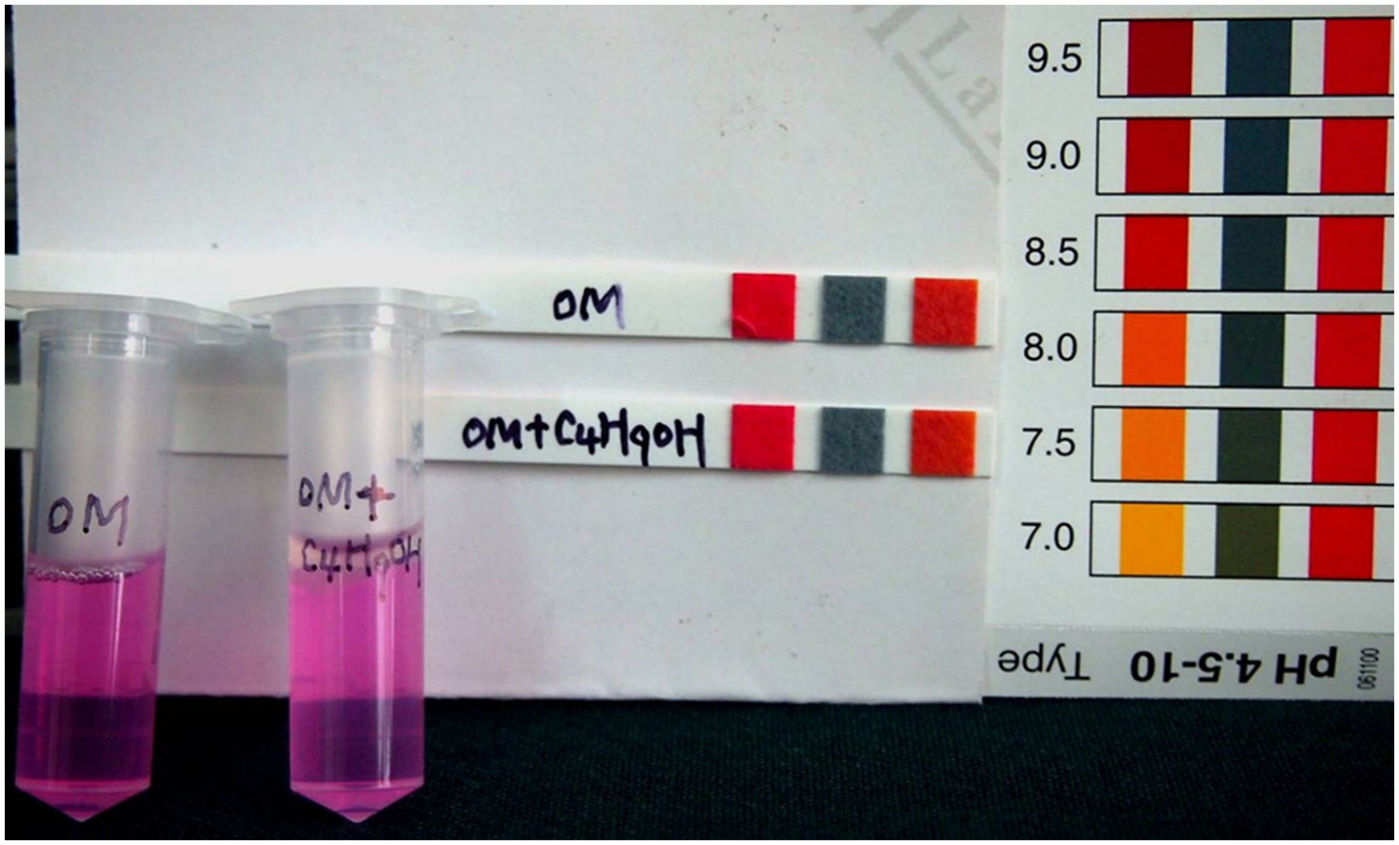
The inactivated FMDV antigen alone or mixed with n-butyl alcohol, the pH values of antigen and mixture are unchanged.

It was also shown that, in most cases, the inactivated antigen could be easily extracted from oil-emulsion vaccines with n-butyl alcohol in just a few minutes without affecting the pH values of the samples (Fig 5). The extracted antigen could then be subsequently quantified using our method. Occasionally, additional n-butyl alcohol (Fig 1G) and a separation funnel were required to achieve a better separation of the aqueous phase. Otherwise, the oil interference would not have been completely minimized (Fig 1F), and the results would have had a higher variability (such as that observed for samples O-5 and O-6).

The OD_490_ values obtained in the assay were directly dependent on the concentrations of the 146S antigen, which have been demonstrated to correlate with the protection level of the FMD vaccine [25–28]. The 146S antigen content of the test samples could be easily calculated using specific pre-programmed EXCEL procedures based on Curve Expert 1.4. Furthermore, the original 146S values of the samples calculated in this way were consistent even over several serial dilutions (4 to 6), suggesting the high precision of the DAS ELISA (Table 2). The within-assay and between-assay variations were acceptable (%CV 2.87–12.43 and 4.33–8.67, respectively), indicating that the DAS ELISA was stable. Therefore, the standard antigen served as a “ruler” between the OD_490_ values and the concentrations of the 146S antigen.

The previous studies indicated that there was a close correlation between the 146S antigen content of the tissue cultures used to prepare the vaccines and the antibody responses against FMD as measured by ELISA and NVT [11, 25–28]. There was also a close correlation between the antibody response and protection for challenge tests [25–28]. So, quality control must be focused on ensuring the consistency of different batches of FMD vaccines by using easier and more practical methods. This ELISA method fitted it rightly. Fig 4 showed that the vaccine 146S antigen concentration was quite variable (Samples 21 to 38). And vaccines with low 146S antigen content can be rejected out *via* this ELISA method, like Sample 27 and Sample 33.

The results indicated that it is possible to quantify the FMDV 146S antigen in samples using a polyclonal antibody. Several previous studies confirmed the feasibility of the DAS ELISA method using a MAb, and there was strict consistency between the MAb-based DAS ELISA and polyclonal antibody-based DAS ELISA. Moreover, comparisons of the antibody preparation and antigen spectrum recognized by the antibody demonstrated that using a polyclonal antibody had an advantage over MAbs. Hence, our DAS ELISA using the polyclonal antibody is credible, simple and conclusive. The 146S content of the standard antigen determined by the SDG method was in agreement with the assessment using the nonlinear standard curves. This finding demonstrated that the FMD antigens and vaccines were similar to the standard antigen. This study provides useful information regarding the quantitative detection of other antigens of interest without the use of animal-based *in vivo* potency testing.

## Conclusion

In conclusion, the DAS ELISA described in this study represents a relatively stable, simple, rapid, inexpensive, sensitive, robust and easy to standardize *in vitro* tool for the quantitative detection of the FMDV 146S antigen in live/inactive viral antigens and oil-emulsion type O vaccines. This alternative method represents a suitable tool that can be used to reduce the use of mice, swine and cattle for FMD vaccine batch control and potency testing. And it still needs to be prospectively validated by analyzing a new vaccine preparation and determining the proper protective dose followed by an *in vivo* vaccination-challenge study to confirm the ELISA findings.

## Supporting Information

S1 File Table A Data used for determining the Limit of Detection (LD). BHK-21 cells (as blank controls) were assayed 20 times in duplicate. LD was evaluated by interpolating the mean of 40 values plus 10 standard deviations. It was 0.155 (0.065+0.009×10 = 0.155). **Table B Data used for determining the Lower Limit of Quantitation (LLQ). Table C Data used for determining the intra- assay precision and the inter-assay precision of the DAS ELISA.** Ten FMDV samples, including 4 vaccine samples (Va1-Va4), 3 antigen samples (Ag1-Ag3) and 3 virus samples (Vi1- Vi3), were assayed 9 times, the measured 146S antigen concentration, the Means, SD and %CVs within an assay and between assays of the samples were showed. **Table D Data used for the specificity of the DAS ELISA (OD**_**490**_). The specificity of the DAS ELISA was analysed through dilution series of the following FMDV strain antigens: O/MYA98, O/China/99, Asia 1/JSL and A/HuBWH serotypes, from a constant high initial amount (2.0 μg/mL), and the blank samples of BHK-21 cells and PBS, as well as the other disease antigens, such as: swine vesicular disease virus (SVDV), classical swine fever virus (CSFV), porcine reproductive and respiratory syndrome virus (PRRSV) and bovine viral diarrhoea virus (BVDV). The results indicated that the O/MYA98 and O/China/99 can be specifically detected among the others by the absorbance values obtained for testing. **Table E 146S antigen quantification samples *via* both DAS ELISA and SDG.** Eighty-five samples including live FMDV (n = 17, 39–55), samples of the inactivated virus preparation (n = 50, 1–20, 576–85) and vaccine samples (n = 18, 21–38) were tested for 146S antigen content with both methods (DAS ELISA and SDG method).
(DOC)
