# Supplementary material for: Quantitative Detection of the Foot-And-Mouth Disease Virus Serotype O 146S Antigen for Vaccine Production Using a Double-Antibody Sandwich ELISA and Nonlinear Standard Curves
Source: PLoS One. 2016 Mar 1;11(3):e0149569. doi: 10.1371/journal.pone.0149569 (PMC4773165; doi:10.1371/journal.pone.0149569)
Supplement: S1 File — Table A Data used for determining the Limit of Detection (LD). BHK-21 cells (as blank controls) were assayed 20 times in duplicate. LD was evaluated by interpolating the mean of 40 values plus 10 standard deviations. It was 0.155 (0.065+0.009×10 = 0.155). Table B Data used for determining the Lower Limit of Quantitation (LLQ). Table C Data used for determining the intra- assay precision and the inter-assay precision of the DAS ELISA. Ten FMDV samples, including 4 vaccine samples (Va1-Va4), 3 antigen samples (Ag1-Ag3) and 3 virus samples (Vi1- Vi3), were assayed 9 times, the measured 146S antigen concentration, the Means, SD and %CVs within an assay and between assays of the samples were showed. Table D Data used for the specificity of the DAS ELISA (OD490). The specificity of the DAS ELISA was analysed through dilution series of the following FMDV strain antigens: O/MYA98, O/China/99, Asia 1/JSL and A/HuBWH serotypes, from a constant high initial amount (2.0 μg/mL), and the blank samples of BHK-21 cells and PBS, as well as the other disease antigens, such as: swine vesicular disease virus (SVDV), classical swine fever virus (CSFV), porcine reproductive and respiratory syndrome virus (PRRSV) and bovine viral diarrhoea virus (BVDV). The results indicated that the O/MYA98 and O/China/99 can be specifically detected among the others by the absorbance values obtained for testing. Table E 146S antigen quantification samples via both DAS ELISA and SDG. Eighty-five samples including live FMDV (n = 17, 39–55), samples of the inactivated virus preparation (n = 50, 1–20, 576–85) and vaccine samples (n = 18, 21–38) were tested for 146S antigen content with both methods (DAS ELISA and SDG method). (DOC) [file pone.0149569.s001.doc]

**Table A Data used for determining the Limit of Detection (LD)**

| **Times** | **BHK-21** | **BHK-21** | **Times** | **BHK-21** | **BHK-21** |
| --- | --- | --- | --- | --- | --- |
| **1** | **0.057** | **0.063** | **11** | **0.077** | **0.078** |
| **2** | **0.055** | **0.058** | **12** | **0.080** | **0.084** |
| **3** | **0.053** | **0.068** | **13** | **0.072** | **0.071** |
| **4** | **0.056** | **0.063** | **14** | **0.075** | **0.065** |
| **5** | **0.053** | **0.058** | **15** | **0.064** | **0.078** |
| **6** | **0.078** | **0.055** | **16** | **0.062** | **0.057** |
| **7** | **0.052** | **0.057** | **17** | **0.061** | **0.058** |
| **8** | **0.067** | **0.070** | **18** | **0.079** | **0.057** |
| **9** | **0.066** | **0.062** | **19** | **0.073** | **0.077** |
| **10** | **0.061** | **0.067** | **20** | **0.082** | **0.058** |

Mean=0.065, S.D=0.0090; BHK-21 cells (as blank controls) were assayed 20 times in duplicate.The limit of detection (LD) was evaluated by interpolating the mean of 40 values of blank controls of BHK-21 cells plus 10 standard deviations. It was 0.155 (0.065+0.009×10=0.155).

**Table B Data used for determining the Lower Limit of Quantitation (LLQ)**

| **2 fold dilution** | **standard antigen OD_490_** | **standard antigen concentration (μg/mL)** |
| --- | --- | --- |
|  | **2.354** | **2.0000** |
| **1:2** | **2.154** | **1.0000** |
| **1:4** | **1.741** | **0.5000** |
| **1:8** | **1.245** | **0.2500** |
| **1:16** | **0.848** | **0.1250** |
| **1:32** | **0.527** | **0.0625** |
| **1:64** | **0.316** | **0.0313** |
| **1:128** | **0.201** | **0.0156** |

The limit of quantitation (LQ）was calculated by the LD (0.155) plus the highest value obtained from the Asia 1/JSL sample (0.311, higher than the values obtained from the A/HuBWH serotype, 0,210), LQ was 0.466. So LLQ was 0.06 µg/mL (0.527>0.466).

**Table C Data used for determining the intra- assay precision and the inter- assay precision of the DAS ELISA**

| **Replications** | **1** | **2** | **3** | **4** | **5** | **intra- assay** | | |
| --- | --- | --- | --- | --- | --- | --- | --- | --- |
| **1#** | **Va-1** ^a^ | **Va-1** | **Va-1** | **Va-1** | **Va-1** | **MEAN** | **SD** ^d^ | **CV %** ^e^ |
| 1 | 2.206 | 1.976 | 1.971 | 2.355 | 2.010 | 2.103 | 0.153 | 7.251 |
| 2 | 2.542 | 1.961 | 2.358 | 2.206 | 2.398 | 2.293 | 0.197 | 8.611 |
| 3 | 2.066 | 2.172 | 1.963 | 2.048 | 1.946 | 2.039 | 0.081 | 3.976 |
| 4 | 1.943 | 1.926 | 1.929 | 1.912 | 2.102 | 1.963 | 0.070 | 3.582 |
| 5 | 1.943 | 2.037 | 2.133 | 1.926 | 1.944 | 1.997 | 0.078 | 3.931 |
| 6 | 1.943 | 2.028 | 2.170 | 1.919 | 2.023 | 2.017 | 0.088 | 4.346 |
| 7 | 2.137 | 2.160 | 1.862 | 1.895 | 1.948 | 2.000 | 0.124 | 6.214 |
| 8 | 2.171 | 1.997 | 1.853 | 1.844 | 1.989 | 1.970 | 0.119 | 6.047 |
| 9 | 1.864 | 1.853 | 1.890 | 1.794 | 1.699 | 1.820 | 0.068 | 3.746 |
|  | P value | 0.1202 |  | **inter-assay** | | **2.022** | **0.119** | **5.909** |
|  |  |  |  |  |  |  |  |  |
| **2#** | **Va-2** | **Va-2** | **Va-2** | **Va-2** | **Va-2** | **MEAN** | **SD** | **CV %** |
| 1 | 3.402 | 3.700 | 3.727 | 3.590 | 3.659 | 3.616 | 0.117 | 3.224 |
| 2 | 3.155 | 3.177 | 2.995 | 3.438 | 3.236 | 3.200 | 0.143 | 4.474 |
| 3 | 3.601 | 3.435 | 3.579 | 3.050 | 3.362 | 3.405 | 0.199 | 5.839 |
| 4 | 3.767 | 3.601 | 3.806 | 3.498 | 3.684 | 3.671 | 0.112 | 3.048 |
| 5 | 3.768 | 3.125 | 3.772 | 3.501 | 3.303 | 3.494 | 0.255 | 7.302 |
| 6 | 3.552 | 3.587 | 3.712 | 3.686 | 3.335 | 3.574 | 0.134 | 3.742 |
| 7 | 3.406 | 3.614 | 3.470 | 3.285 | 3.316 | 3.418 | 0.118 | 3.446 |
| 8 | 3.696 | 3.791 | 3.655 | 3.754 | 3.220 | 3.623 | 0.207 | 5.713 |
| 9 | 3.792 | 3.449 | 3.722 | 3.768 | 3.834 | 3.713 | 0.137 | 3.681 |
|  | P value | 0.0785 |  | **inter-assay** | | **3.524** | **0.152** | **4.326** |
|  |  |  |  |  |  |  |  |  |
| **3#** | **Va-3** | **Va -3** | **Va -3** | **Va -3** | **Va -3** | **MEAN** | **SD** | **CV %** |
| 1 | 8.049 | 6.761 | 7.785 | 8.304 | 8.656 | 7.911 | 0.643 | 8.125 |
| 2 | 11.205 | 9.595 | 10.872 | 9.920 | 10.912 | 10.501 | 0.626 | 5.962 |
| 3 | 7.813 | 8.077 | 9.331 | 10.350 | 7.853 | 8.685 | 1.001 | 11.527 |
| 4 | 10.097 | 8.692 | 9.497 | 9.773 | 9.299 | 9.472 | 0.474 | 4.999 |
| 5 | 8.420 | 9.442 | 10.326 | 8.424 | 10.388 | 9.400 | 0.866 | 9.213 |
| 6 | 9.068 | 8.270 | 8.154 | 8.680 | 8.898 | 8.614 | 0.352 | 4.091 |
| 7 | 7.202 | 7.315 | 7.777 | 8.624 | 8.898 | 7.963 | 0.685 | 8.601 |
| 8 | 9.232 | 8.808 | 8.405 | 9.595 | 10.248 | 9.257 | 0.636 | 6.875 |
| 9 | 9.118 | 8.918 | 8.210 | 8.606 | 7.784 | 8.527 | 0.482 | 5.649 |
|  | P value | 0.0504 |  | **inter-assay** | | **8.926** | **0.774** | **8.666** |
|  |  |  |  |  |  |  |  |  |
| **4#** | **Va -4** | **Va -4** | **Va -4** | **Va -4** | **Va -4** | **MEAN** | **SD** | **CV %** |
| 1 | 20.081 | 24.104 | 20.527 | 21.393 | 21.907 | 21.602 | 1.405 | 6.502 |
| 2 | 20.017 | 21.351 | 20.382 | 21.180 | 21.982 | 20.983 | 0.702 | 3.347 |
| 3 | 23.866 | 21.478 | 26.058 | 25.625 | 24.104 | 24.226 | 1.613 | 6.659 |
| 4 | 20.110 | 20.103 | 22.818 | 21.119 | 19.889 | 20.808 | 1.092 | 5.249 |
| 5 | 21.845 | 21.909 | 23.094 | 19.162 | 26.975 | 22.597 | 2.540 | 11.240 |
| 6 | 19.149 | 21.301 | 23.091 | 20.981 | 23.844 | 21.673 | 1.656 | 7.640 |
| 7 | 24.473 | 24.326 | 23.551 | 23.115 | 22.560 | 23.605 | 0.722 | 3.059 |
| 8 | 21.830 | 19.948 | 22.330 | 21.464 | 25.922 | 22.299 | 1.978 | 8.872 |
| 9 | 18.426 | 20.587 | 20.068 | 22.142 | 17.483 | 19.741 | 1.638 | 8.297 |
|  | P value | 0.0932 |  | **inter-assay** | | **21.948** | **1.324** | **6.033** |
|  |  |  |  |  |  |  |  |  |
| **5#** | **Ag-1** ^b^ | **Ag-1** | **Ag-1** | **Ag-1** | **Ag-1** | **MEAN** | **SD** | **CV %** |
| 1 | 58.189 | 59.764 | 56.458 | 49.837 | 53.368 | 55.523 | 3.548 | 6.390 |
| 2 | 50.258 | 46.770 | 44.541 | 45.623 | 48.854 | 47.209 | 2.089 | 4.426 |
| 3 | 54.506 | 59.324 | 58.324 | 45.488 | 45.802 | 52.689 | 5.972 | 11.335 |
| 4 | 57.403 | 61.038 | 60.038 | 58.803 | 62.038 | 59.864 | 1.632 | 2.727 |
| 5 | 47.100 | 48.966 | 52.326 | 55.006 | 45.534 | 49.786 | 3.455 | 6.939 |
| 6 | 48.409 | 57.462 | 47.206 | 50.752 | 50.395 | 50.845 | 3.555 | 6.991 |
| 7 | 48.907 | 49.043 | 43.399 | 51.590 | 51.685 | 48.925 | 3.009 | 6.150 |
| 8 | 46.181 | 51.155 | 45.777 | 49.519 | 55.803 | 49.687 | 3.665 | 7.377 |
| 9 | 48.835 | 52.369 | 50.395 | 51.685 | 43.490 | 49.355 | 3.171 | 6.424 |
|  | P value | 0.0545 |  | **inter-assay** | | **51.543** | **3.709** | **7.197** |
|  |  |  |  |  |  |  |  |  |
| **6#** | **Ag-2** | **Ag-2** | **Ag-2** | **Ag-2** | **Ag-2** | **MEAN** | **SD** | **CV %** |
| 1 | 8.749 | 9.061 | 8.485 | 9.004 | 9.356 | 8.931 | 0.295 | 3.302 |
| 2 | 8.344 | 8.466 | 7.394 | 8.480 | 8.301 | 8.197 | 0.407 | 4.967 |
| 3 | 8.060 | 8.255 | 8.157 | 8.748 | 7.423 | 8.129 | 0.425 | 5.227 |
| 4 | 8.125 | 8.213 | 9.420 | 8.031 | 7.965 | 8.351 | 0.541 | 6.482 |
| 5 | 7.402 | 8.768 | 9.618 | 8.303 | 8.484 | 8.515 | 0.717 | 8.415 |
| 6 | 8.303 | 8.849 | 7.513 | 8.377 | 8.122 | 8.233 | 0.433 | 5.255 |
| 7 | 8.068 | 7.587 | 7.776 | 8.105 | 8.698 | 8.047 | 0.377 | 4.690 |
| 8 | 7.705 | 8.464 | 7.402 | 8.768 | 7.423 | 7.952 | 0.561 | 7.049 |
| 9 | 7.560 | 9.574 | 9.160 | 8.107 | 8.405 | 8.561 | 0.724 | 8.452 |
|  | P value | 0.2372 |  | **inter-assay** | | **8.324** | **0.591** | **7.104** |
|  |  |  |  |  |  |  |  |  |
| **7#** | **Ag-3** | **Ag-3** | **Ag-3** | **Ag-3** | **Ag-3** | **MEAN** | **SD** | **CV %** |
| 1 | 0.534 | 0.552 | 0.553 | 0.521 | 0.511 | 0.534 | 0.017 | 3.096 |
| 2 | 0.519 | 0.543 | 0.565 | 0.583 | 0.571 | 0.556 | 0.022 | 4.044 |
| 3 | 0.557 | 0.546 | 0.526 | 0.566 | 0.523 | 0.544 | 0.017 | 3.116 |
| 4 | 0.507 | 0.515 | 0.522 | 0.571 | 0.505 | 0.524 | 0.024 | 4.609 |
| 5 | 0.525 | 0.568 | 0.584 | 0.568 | 0.565 | 0.562 | 0.020 | 3.531 |
| 6 | 0.521 | 0.541 | 0.559 | 0.581 | 0.552 | 0.551 | 0.020 | 3.606 |
| 7 | 0.462 | 0.505 | 0.535 | 0.547 | 0.513 | 0.512 | 0.029 | 5.734 |
| 8 | 0.467 | 0.472 | 0.463 | 0.458 | 0.497 | 0.471 | 0.014 | 2.875 |
| 9 | 0.460 | 0.484 | 0.513 | 0.568 | 0.540 | 0.513 | 0.039 | 7.523 |
|  | P value | 0.0612 |  | **inter-assay** | | **0.530** | **0.027** | **5.038** |
|  |  |  |  |  |  |  |  |  |
| **8#** | **Vi-1** ^c^ | **Vi-1** | **Vi-1** | **Vi-1** | **Vi-1** | **MEAN** | **SD** | **CV %** |
| 1 | 1.238 | 1.307 | 1.301 | 1.348 | 1.475 | 1.334 | 0.079 | 5.920 |
| 2 | 1.242 | 1.313 | 1.518 | 1.349 | 1.548 | 1.394 | 0.119 | 8.539 |
| 3 | 1.460 | 1.506 | 1.729 | 1.372 | 1.177 | 1.449 | 0.180 | 12.430 |
| 4 | 1.440 | 1.345 | 1.544 | 1.203 | 1.413 | 1.389 | 0.113 | 8.125 |
| 5 | 1.491 | 1.384 | 1.213 | 1.465 | 1.251 | 1.361 | 0.112 | 8.208 |
| 6 | 1.365 | 1.302 | 1.437 | 1.510 | 1.500 | 1.423 | 0.079 | 5.584 |
| 7 | 1.640 | 1.575 | 1.689 | 1.627 | 1.715 | 1.649 | 0.049 | 2.966 |
| 8 | 1.379 | 1.343 | 1.545 | 1.529 | 1.615 | 1.482 | 0.104 | 6.986 |
| 9 | 1.464 | 1.461 | 1.104 | 1.532 | 1.202 | 1.353 | 0.092 | 6.818 |
|  | P value | 0.1236 |  | **inter-assay** | | **1.426** | **0.091** | **6.362** |
|  |  |  |  |  |  |  |  |  |
| **9#** | **Vi-2** | **Vi-2** | **Vi-2** | **Vi-2** | **Vi-2** | **MEAN** | **SD** | **CV %** |
| 1 | 2.143 | 2.483 | 2.211 | 2.048 | 2.124 | 2.202 | 0.150 | 6.814 |
| 2 | 2.075 | 2.277 | 2.092 | 1.997 | 1.903 | 2.069 | 0.124 | 5.973 |
| 3 | 2.397 | 2.290 | 2.432 | 2.158 | 2.372 | 2.330 | 0.098 | 4.198 |
| 4 | 2.134 | 2.515 | 2.260 | 2.214 | 2.375 | 2.300 | 0.133 | 5.786 |
| 5 | 2.034 | 2.571 | 2.149 | 2.210 | 2.064 | 2.206 | 0.193 | 8.759 |
| 6 | 2.150 | 2.386 | 2.428 | 2.320 | 2.067 | 2.270 | 0.139 | 6.114 |
| 7 | 2.496 | 2.419 | 2.493 | 2.406 | 2.215 | 2.406 | 0.102 | 4.252 |
| 8 | 2.638 | 2.358 | 2.532 | 2.432 | 2.285 | 2.449 | 0.125 | 5.110 |
| 9 | 2.373 | 2.406 | 2.234 | 2.182 | 2.272 | 2.293 | 0.084 | 3.664 |
|  | P value | 0.0759 |  | **inter-assay** | | **2.280** | **0.107** | **4.707** |
|  |  |  |  |  |  |  |  |  |
| **10#** | **Vi-3** | **Vi-3** | **Vi-3** | **Vi-3** | **Vi-3** | **MEAN** | **SD** | **CV %** |
| 1 | 2.684 | 2.707 | 3.096 | 2.623 | 2.853 | 2.793 | 0.169 | 6.068 |
| 2 | 2.604 | 3.019 | 2.866 | 2.865 | 2.620 | 2.795 | 0.159 | 5.701 |
| 3 | 2.816 | 2.779 | 3.000 | 2.717 | 2.902 | 2.843 | 0.099 | 3.469 |
| 4 | 3.004 | 2.725 | 2.922 | 2.607 | 2.741 | 2.800 | 0.143 | 5.117 |
| 5 | 3.612 | 3.447 | 3.582 | 3.594 | 2.934 | 3.434 | 0.257 | 7.473 |
| 6 | 2.667 | 2.902 | 2.796 | 2.616 | 3.429 | 2.882 | 0.291 | 10.105 |
| 7 | 2.645 | 2.996 | 2.973 | 2.893 | 2.751 | 2.852 | 0.135 | 4.719 |
| 8 | 2.797 | 2.939 | 2.902 | 2.934 | 3.234 | 2.961 | 0.146 | 4.918 |
| 9 | 2.892 | 2.958 | 2.628 | 2.807 | 3.334 | 2.924 | 0.233 | 7.975 |
|  | P value | 0.0883 |  | **inter-assay** | | **2.920** | **0.190** | **6.499** |
| **inter-assay** **:P value=0.0772** | | | | | | | | |

^a^ Va=Vaccine; ^b^ Ag=Antigen;^c^ Vi=Virus; ^d^ SD= Standard Deviation; ^e^ CV= Coefficient of Variation

**Table D Data used for the specificity of the DAS ELISA (OD_490_)**

| **Dilution** | **O/China/99** | **O/MYA98** | **A/HuBWH** | **Asia 1/JS** | **BHK** | **SVDV** | **CSFV** | **PRRSV** | **BVDV** | **PRRSV** | **PBS** |
| --- | --- | --- | --- | --- | --- | --- | --- | --- | --- | --- | --- |
| 1/1 | 2.354 | 2.412 | 0.210 | 0.311 | 0.057 | 0.053 | 0.065 | 0.063 | 0.060 | 0.063 | 0.053 |
| 1/2 | 2.154 | 2.228 | 0.194 | 0.233 | 0.055 | 0.051 | 0.051 | 0.055 | 0.058 | 0.055 | 0.058 |
| 1/4 | 1.741 | 1.891 | 0.166 | 0.169 | 0.053 | 0.047 | 0.049 | 0.048 | 0.055 | 0.048 | 0.052 |
| 1/8 | 1.245 | 1.428 | 0.140 | 0.124 | 0.056 | 0.048 | 0.046 | 0.048 | 0.058 | 0.048 | 0.050 |
| 1/16 | 0.848 | 0.975 | 0.095 | 0.098 | 0.057 | 0.047 | 0.045 | 0.043 | 0.049 | 0.043 | 0.062 |
| 1/32 | 0.527 | 0.577 | 0.077 | 0.076 | 0.058 | 0.051 | 0.056 | 0.053 | 0.050 | 0.053 | 0.060 |
| 1/64 | 0.316 | 0.358 | 0.066 | 0.063 | 0.068 | 0.054 | 0.060 | 0.075 | 0.055 | 0.075 | 0.080 |
| 1/128 | 0.201 | 0.235 | 0.061 | 0.055 | 0.063 | 0.058 | 0.063 | 0.067 | 0.050 | 0.067 | 0.062 |

**Table E 146S antigen quantification samples *via*** both DAS ELISA and SDG

|  |  | **NAMES** | **ELISA** | **SDG** |  |  | **NAMES** | **ELISA** | **SDG** |  |  | **NAMES** | **ELISA** | **SDG** |
| --- | --- | --- | --- | --- | --- | --- | --- | --- | --- | --- | --- | --- | --- | --- |
| 1 | Ag | **6-1** | **2.389** | **2.498** | 31 | Va | **EV-1** | **2.760** | **2.159** | 61 | Ag | **BO-1** | **2.009** | **1.439** |
| 2 | Ag | **23-1** | **1.076** | **1.032** | 32 | Va | **EV-2** | **2.910** | **2.369** | 62 | Ag | **AO-1** | **1.268** | **1.229** |
| 3 | Ag | **34-1** | **1.486** | **0.835** | 33 | Va | **DV**-2 | **1.070** | **1.193** | 63 | Ag | **OM1345** | **2.010** | **1.780** |
| 4 | Ag | **A-25** | **1.983** | **1.333** | 34 | Va | **O-1** | **2.494** | **1.100** | 64 | Ag | **B13006③** | **2.575** | **3.101** |
| 5 | Ag | **A-26** | **2.420** | **1.874** | 35 | Va | **O-2** | **4.631** | **5.130** | 65 | Ag | **B13006④** | **2.796** | **2.984** |
| 6 | Ag | **16-1** | **23.937** | **19.832** | 36 | Va | **O-4** | **1.691** | **1.710** | 66 | Ag | **A13001③** | **2.300** | **2.954** |
| 7 | Ag | **A-28** | **2.141** | **1.618** | 37 | Va | **O-5** | **2.861** | **2.860** | 67 | Ag | **A13001④** | **2.422** | **2.837** |
| 8 | Ag | **A-29** | **2.184** | **2.027** | 38 | Va | **O-6** | **1.975** | **1.890** | 68 | Ag | **A13002③** | **2.213** | **2.340** |
| 9 | Ag | **YFBY1** | **2.555** | **2.580** | 39 | Vi | **B13006①** | **1.359** | **1.814** | 69 | Ag | **A13002④** | **2.233** | **2.837** |
| 10 | Ag | **YFBY2** | **5.655** | **4.581** | 40 | Vi | **B13006②** | **1.257** | **1.141** | 70 | Ag | **B13001③** | **2.714** | **2.282** |
| 11 | Ag | **YFBY4** | **4.253** | **4.528** | 41 | Vi | **A13001①** | **2.553** | **1.755** | 71 | Ag | **B13001④** | **2.448** | **2.106** |
| 12 | Ag | **B-26** | **3.655** | **2.734** | 42 | Vi | **A13001②** | **1.314** | **1.053** | 72 | Ag | **B13002③** | **2.201** | **1.931** |
| 13 | Ag | **B-28** | **2.793** | **2.317** | 43 | Vi | **A13002①** | **2.382** | **1.170** | 73 | Ag | **B13002④** | **2.229** | **1.784** |
| 14 | Ag | **B-29** | **2.965** | **2.071** | 44 | Vi | **A13002②** | **1.351** | **0.936** | 74 | Ag | **B13005③** | **2.646** | **1.784** |
| 15 | Ag | **B-30** | **2.779** | **2.267** | 45 | Vi | **B13001①** | **2.946** | **2.486** | 75 | Ag | **B13005④** | **2.341** | **1.638** |
| 16 | Ag | **47-1** | **2.377** | **1.814** | 46 | Vi | **B13001②** | **1.318** | **1.375** | 76 | Ag | **YFBY 3** | **3.386** | **4.358** |
| 17 | Ag | **ZM2** | **5.940** | **3.405** | 47 | Vi | **B13002①** | **2.932** | **1.784** | 77 | Ag | **B99-3** | **2.000** | **2.597** |
| 18 | Ag | **M3** | **2.809** | **1.931** | 48 | Vi | **B13002②** | **1.427** | **1.316** | 78 | Ag | **B99-4** | **1.974** | **2.738** |
| 19 | Ag | **ZM3** | **5.185** | **3.317** | 49 | Vi | **B13005①** | **2.693** | **2.428** | 79 | Ag | **B98-3** | **2.664** | **3.054** |
| 20 | Ag | **AO-3** | **0.517** | **0.369** | 50 | Vi | **B13005②** | **0.934** | **1.784** | 80 | Ag | **B98-4** | **2.455** | **2.896** |
| 21 | Va | **YFBM1** | **3.406** | **3.124** | 51 | Vi | **B98-1** | **3.363** | **2.317** | 81 | Ag | **A-30** | **2.085** | **1.872** |
| 22 | Va | **YFBM2** | **6.667** | **5.897** | 52 | Vi | **B98-2** | **1.620** | **1.615** | 82 | Ag | **A-24** | **1.599** | **1.258** |
| 23 | Va | **YFBM3** | **8.485** | **9.266** | 53 | Vi | **B99-1** | **1.191** | **2.229** | 83 | Ag | **A-34** | **2.531** | **1.831** |
| 24 | Va | **YFBM4** | **4.354** | **5.177** | 54 | Vi | **B99-2** | **2.432** | **2.633** | 84 | Ag | **B-24** | **2.409** | **2.065** |
| 25 | Va | **F13104** | **2.102** | **1.784** | 55 | Vi | **YFB700** | **2.581** | **1.439** | 85 | Ag | **B-34** | **2.555** | **2.200** |
| 26 | Va | **F13105** | **2.048** | **2.311** | 56 | Ag | **EO-1** | **5.287** | **3.422** |  |  |  |  |  |
| 27 | Va | **F13106** | **1.405** | **1.404** | 57 | Ag | **EO-2** | **5.290** | **3.879** |  |  |  |  |  |
| 28 | Va | **F13107** | **2.561** | **2.516** | 58 | Ag | **CO-1** | **3.292** | **2.036** |  |  |  |  |  |
| 29 | Va | **F13108** | **2.054** | **1.989** | 59 | Ag | **CO-2** | **2.949** | **1.860** |  |  |  |  |  |
| 30 | Va | **F13101** | **2.173** | **2.165** | 60 | Ag | **DO-1** | **2.466** | **2.755** |  |  |  |  |  |
